# Supplementary material for: Causal modeling of gene effects from regulators to programs to traits: integration of genetic associations and Perturb-seq
Source: bioRxiv. 2025 Jan 24:2025.01.22.634424. Preprint. [Version 1] doi: 10.1101/2025.01.22.634424 (PMC11785173; doi:10.1101/2025.01.22.634424)
Supplement: Supplement 3 [file NIHPP2025.01.22.634424v1-supplement-3.pdf]

bioRxiv preprint doi: https://doi.org/10.1101/2025.01.22.634424; this version posted January 24, 2025. The copyright holder for this preprint (which was not certified by peer review) is the author/funder, who has granted bioRxiv a license to display the preprint in perpetuity. It is made available under aCC-BY 4.0 International license.

Supplementary Material

Supplementary Notes

GWAS enrichment to gene regulatory networks from Perturb-seq.

Previous studies have investigated the enrichment of GWAS signals to the programs and regulators identified by Perturb-seq [28–30] (and related papers [27, 31]). Consistent with our GWAS analysis here, the previous studies that aimed to link GWAS to Perturb-seq have generally found significant enrichment at the program level, but limited enrichment of regulators. We discuss some of the key papers here:

A pioneering study by Schnitzler *et al.* [28] tested the enrichment of coronary artery disease GWAS variants to programs and regulators identified from Perturb-seq in telomerase-immortalized human aortic endotherial cells, where they perturbed 1,661 genes close to GWAS hits. They applied cNMF to their dataset and identified five out of 50 programs for which the union of program and regulator genes showed significant enrichment to GWAS-linked genes. Their signal was primarily driven by GWAS hits at the program level, and in total only six regulators overlapped GWAS-linked genes for the five enriched programs. Using relationships between regulators and programs inferred from the Perturb-seq data, they proposed a regulatory pathway that controls key programs, but this only contained two GWAS-linked genes. While the authors successfully validated the roles of these key regulators (*CCM2* and *TLNRD1*) in atherosclerosis etiology, it may be difficult to generalize this approach to connect regulatory pathways to traits at genome-scale.

Yao *et al.* [30] performed Perturb-seq in THP1 cells with or without LPS stimulation, where they perturbed 598 genes in a cell-pooled experiment. They identified three modules of regulators that had similar effects on transcriptional programs. One module (genes that suppress the LPS response) was found to be enriched for polygenic signals for 2 out of 64 tested traits (lymphocyte and neutrophil percentages) in an analysis using sc-linker [94]. Positive regulators of the LPS response did not show enrichment to any of the traits. In contrast, they observed many examples of downstream gene signatures (genes whose expression is affected by KD of a focal gene) that were enriched to various traits/ diseases.

Geiger-Schuller *et al.* [29] performed Perturb-seq in mouse primary dendritic cells, targeting 1,130 genes associated with E3 ligases. They tested the enrichment of modules of regulators to polygenic risk of 60 traits/diseases with sc-linker [94] and MAGMA [95], and they reported that one regulator gene module was enriched across all traits, with relatively higher enrichment to immune mediated diseases. In contrast, program genes showed more trait-specific enrichment patterns.

Although these studies used different experimental and analytical protocols, their observations have similarities: despite trait-specific enrichment of co-regulated gene programs, program regulators did not show enrichment to GWAS signals, or any enrichment that *was* found was not trait-specific.

We propose that the difficulties in identifying trait-specific regulators from GWAS enrichment analyses arise from two aspects. First, for GWAS signals, enrichment tests cannot account for the direction of association of genes. As observed in the studies above [29,30] and in our analysis,

bioRxiv preprint doi: https://doi.org/10.1101/2025.01.22.634424; this version posted January 24, 2025. The copyright holder for this preprint (which was not certified by peer review) is the author/funder, who has granted bioRxiv a license to display the preprint in perpetuity. It is made available under aCC-BY 4.0 International license.

genes often have regulatory effects on multiple programs with different directions. Accounting for these directional effects may be important not only for gaining power in the enrichment analysis but also for understanding the trait-specific enrichment. Second, the cis-effect size of common variants on gene dosage is expected to be smaller than that of LoF variants. Furthermore, the trans effects of genes are not linear in relation to gene dosage for many genes, a phenomenon known as buffering [64, 65]. Thus, the ranking of GWAS top hits based on their effect size on the trans program may not align with the ranking of the cis-gene's trans effect on the program under the KD experiments. Together, the top hits prioritized by GWAS may not overlap well with the top regulators in Perturb-seq, and LoF burden test could be a good alternative for it.

Negative-feedback and co-regulation across pathways explain RDW associations.

In RDW, the hemoglobin synthesis program exhibited a discordant enrichment pattern in regulator-burden correlation and program burden effects (Figure 4D). Positive program burden effects indicate that the disrupted hemoglobin synthesis process leads to higher RDW. Considering that RDW is defined as the standard deviation of erythroid size, which quantifies the quality of erythrocytes, this observed direction makes biological sense. However, the regulator-burden correlation indicated that up-regulation of hemoglobin synthesis program results in higher RDW. These resulted in seemingly discordant enrichment pattern. How can we interpret this observation?

Here, regulators of the hemoglobin synthesis program also had effects on the ATP-dependent activity program, the other enriched program. However, regulators of the ATP activity program did not have significant effects on the hemoglobin synthesis program, on average (Figure S6D). This pattern suggests a directional relationship from hemoglobin synthesis program activity to ATP activity, corresponding to the biological negative feedback observed between these two pathways [96]. We applied a causal inference method originally used for interpreting GWAS top variant associations [92] to our case (Methods) and indeed observed that the causal model was 10^6 times more likely than the non-causal models. Thus, the regulatory patterns in the Perturb-seq data suggested a negative feedback from the hemoglobin synthesis pathway to the ATP activity pathway.

In such case, regulators of the hemoglobin synthesis program impact on RDW through their effect on ATP activity. It can result in discordant regulator enrichment for the hemoglobin program in the marginal test.

In addition, regulators of the ATP activity and autophagy programs are highly shared. The multiple regression model suggested that autophagy regulation has the largest effect on RDW among these programs (Figure S6D).

In general, when trait-relevant programs are connected by this kind of feedback loop and/or co-regulation, it can result in seemingly discordant enrichment.

Overlap of program genes and regulator genes in the model.

Although not very frequent, some of the genes were both top-loading program genes and significant regulators in the selected model for the trait in the gene-to-program-to-trait map (Figure S9 for MCH). Since we cannot quantify the program gene KD effect on program *function*, unlike its

bioRxiv preprint doi: https://doi.org/10.1101/2025.01.22.634424; this version posted January 24, 2025. The copyright holder for this preprint (which was not certified by peer review) is the author/funder, who has granted bioRxiv a license to display the preprint in perpetuity. It is made available under aCC-BY 4.0 International license.

expression levels, it is difficult to predict the total effects of the gene on the trait and the signs of associations in this case.

In such cases, we prioritized the signs of program burden effects, based on the assumption that the gene effects on program functions are generally larger than their regulatory effects on other programs.

For example, *POLE* was one of the top-loading program genes for the S phase cell cycle program. At the same time, it was a negative regulator for both the hemoglobin synthesis program and the autophagy program. These programs were all associated with MCH.

As the sign of association of the S phase program with MCH, predicted from program burden effects, was repressing, the predicted direction of POLE for MCH as an S phase program gene was "repressing".

On the contrary, the regulator-burden correlation indicated that the quantity of the hemoglobin synthesis program was positively associated with MCH, while the autophagy program was negatively associated. Thus, the regulatory effects of POLE on these two programs canceled each other out, leading to our predicted signs of the effects of POLE on MCH as a regulator being weakly "activating".

In our model, we predicted that its effect on MCH was "repressing" as we prioritize the signs from program burden effects. In this case, it was concordant with the observed γ of this gene on MCH.

In contrast, RDW was only associated with POLE via its regulatory effects on the autophagy program. Also in this case, the sign of association was predicted consistently from the model.

Thus, in this case, the predicted directions of *POLE* associations with the traits were correct for both traits. However, this assumption may not always hold true. For example, a gene co-regulated with other functional program genes may not have direct effects on program functions by itself.

Association of *SLC4A1* with MCH and RDW.

LoF of *SLC4A1* had the strong effects on MCH and RDW in the same direction (Figure 2D), in contrast to the negative genetic correlation of these traits. *SLC4A1* encodes a cell surface protein expressed on erythrocytes. LoF of one copy of this gene is a known cause of hereditary spherocytosis [97]. Spherocytosis leads to the secondary hemolysis, which can increase both RDW and MCH [97]. In this case, this gene affects these traits not through the regulation of other genes. At the same time, a quantitative change in this gene may not be linearly associated with the trait—for instance, a 20% decrease in gene dosage may not result in hemolysis. The function of this type of gene on the trait cannot be investigated solely through Perturb-seq; experimental and clinical knowledge is needed to complement it.

bioRxiv preprint doi: https://doi.org/10.1101/2025.01.22.634424; this version posted January 24, 2025. The copyright holder for this preprint (which was not certified by peer review) is the author/funder, who has granted bioRxiv a license to display the preprint in perpetuity. It is made available under aCC-BY 4.0 International license.

Supplementary Figures.

Supplementary Tables.

Table S1: **Annotation of cNMF programs to biological pathways.**

Table S2: **List of control traits.**

bioRxiv preprint doi: https://doi.org/10.1101/2025.01.22.634424; this version posted January 24, 2025. The copyright holder for this preprint (which was not certified by peer review) is the author/funder, who has granted bioRxiv a license to display the preprint in perpetuity. It is made available under aCC-BY 4.0 International license.

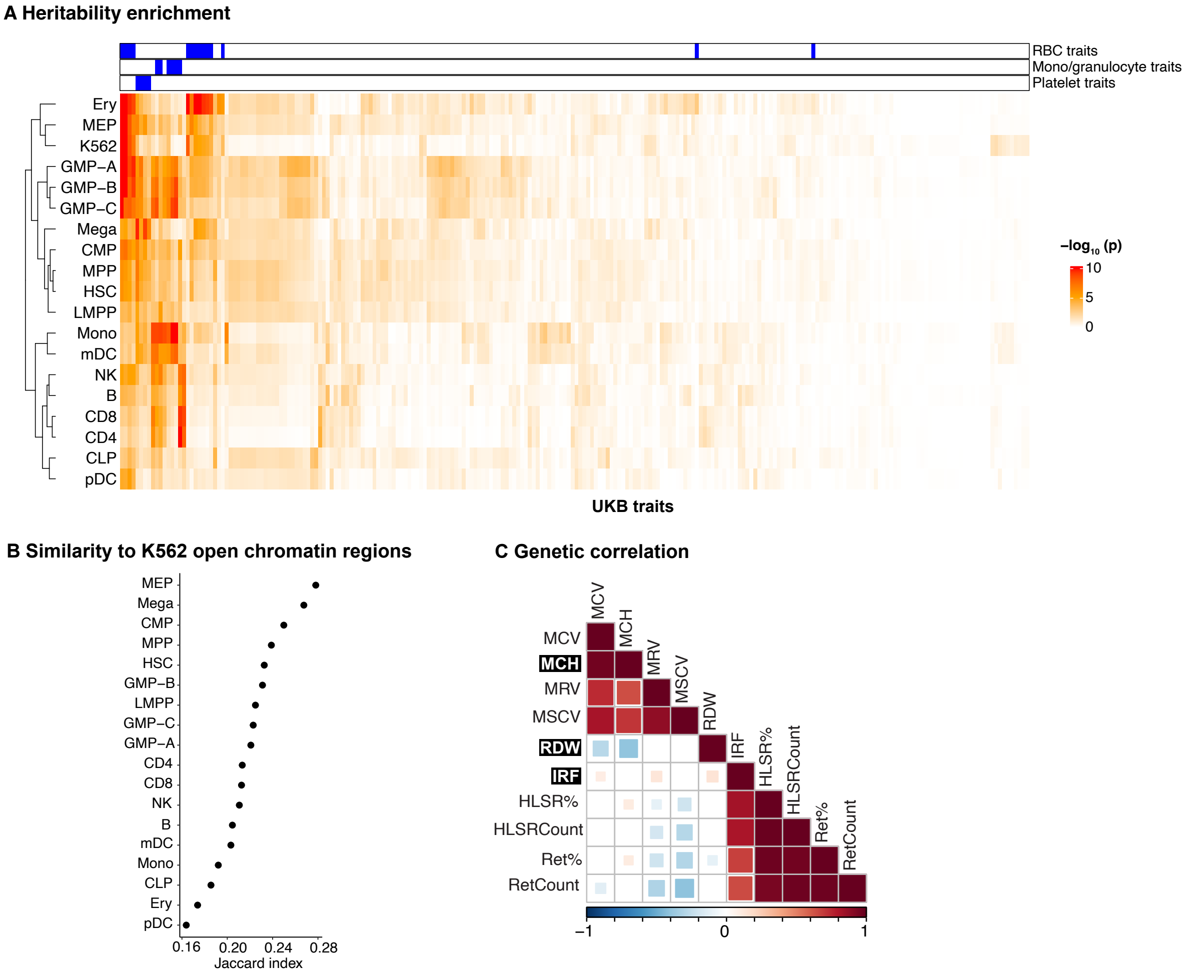

Figure S1: **Analysis of the heritability of multiple traits from GWAS, related to Figure 1.** ***A)*** *Heritability enrichment of UKB traits to 18 primary hematopoietic cell types [75] and K562. Heritability enrichment was estimated with S-LDSC by adding each annotation to the baseline model. Traits associated with the morphology or quantity of RBC, monocyte/granulocyte or platelet are labeled on top. Both cell types (rows) and traits (columns) are hierarchically clustered based on their patterns of enrichment. K562 showed the closest similarity to MEP.* ***B)*** *Similarity of open chromatin regions of primary cell types to K562. Plotted are Jaccard index, which captures the proportion of open chromatin regions that are shared with K562.* ***C)*** *Genetic correlation across traits which were enriched to K562 in S-LDSC analysis.*

bioRxiv preprint doi: https://doi.org/10.1101/2025.01.22.634424; this version posted January 24, 2025. The copyright holder for this preprint (which was not certified by peer review) is the author/funder, who has granted bioRxiv a license to display the preprint in perpetuity. It is made available under aCC-BY 4.0 International license.

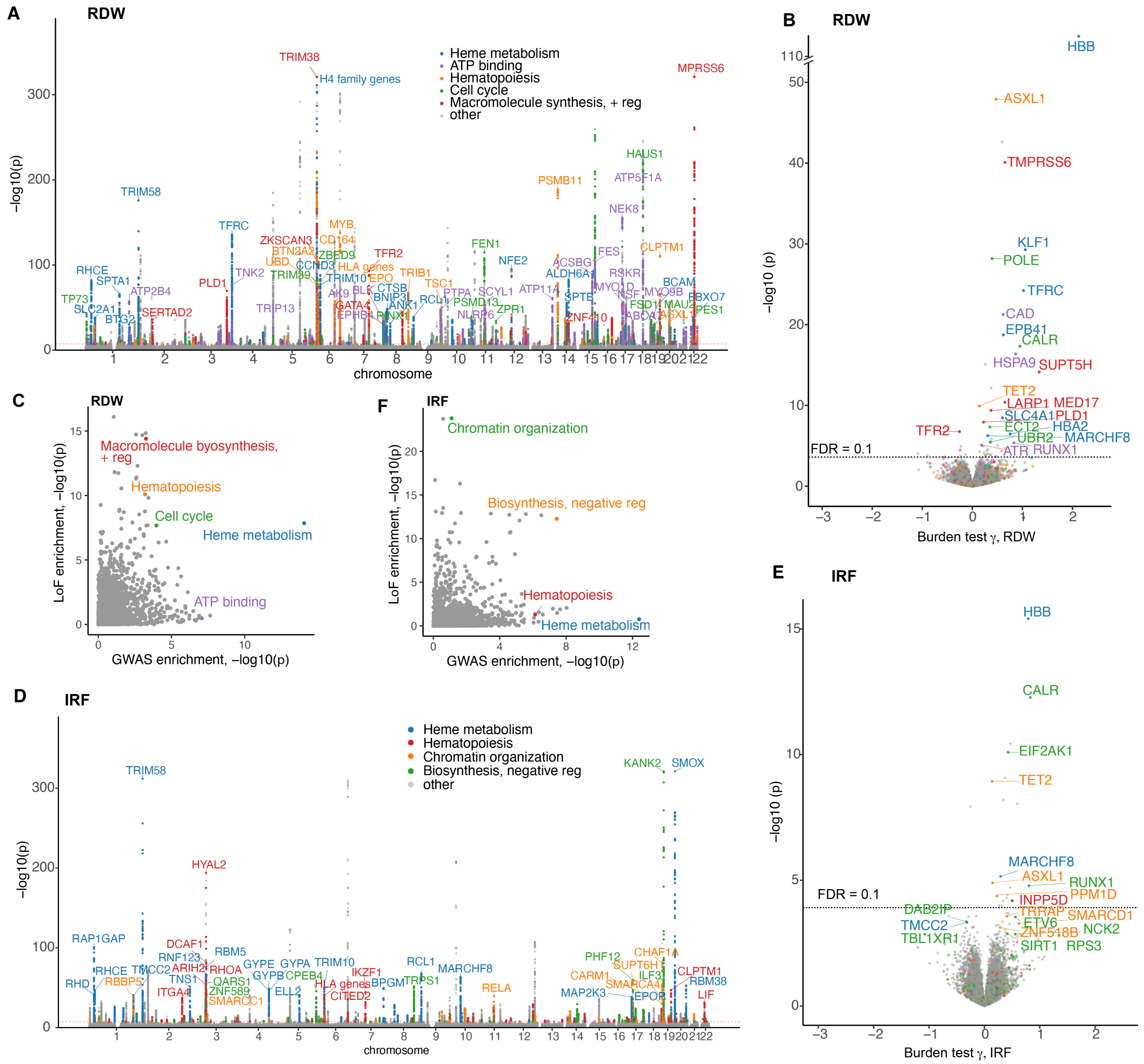

Figure S2: **Pathway enrichments for blood trait associations., related to Figure 2** *A) Genetic associations identified from UKB GWAS for RDW. Variants located within a 100 kbp window centered on the transcription start site of the genes in the gene set are colored.* ***B)*** *Gene association with RDW from UKB LoF burden test. Colors indicate the same gene sets as A). Genes labeled have FDR < 0.01 and belong to the gene sets.* ***C)*** *Pathway enrichment of GWAS and LoF burden test top genes for RDW. For GWAS, the closest genes from the independent top variants were used. For the LoF burden test, genes were ranked by the absolute posterior effect size after GeneBayes, and the same number of genes as in GWAS was used.* ***D)*** *Genetic associations identified from UKB GWAS for IRF. Variants located within a 100 kbp window centered on the transcription start site of the genes in the gene set are colored.* ***E)*** *Gene association with IRF from UKB LoF burden test. Colors indicate the same gene sets as D). Genes labeled have FDR < 0.5 and belong to the gene sets.* ***F)*** *Pathway enrichment of GWAS and LoF burden test top genes for IRF. For GWAS, the closest genes from the independent top variants were used. For the LoF burden test, genes were ranked by the absolute posterior effect size after GeneBayes, and the same number of genes as in GWAS was used.*

bioRxiv preprint doi: https://doi.org/10.1101/2025.01.22.634424; this version posted January 24, 2025. The copyright holder for this preprint (which was not certified by peer review) is the author/funder, who has granted bioRxiv a license to display the preprint in perpetuity. It is made available under aCC-BY 4.0 International license.

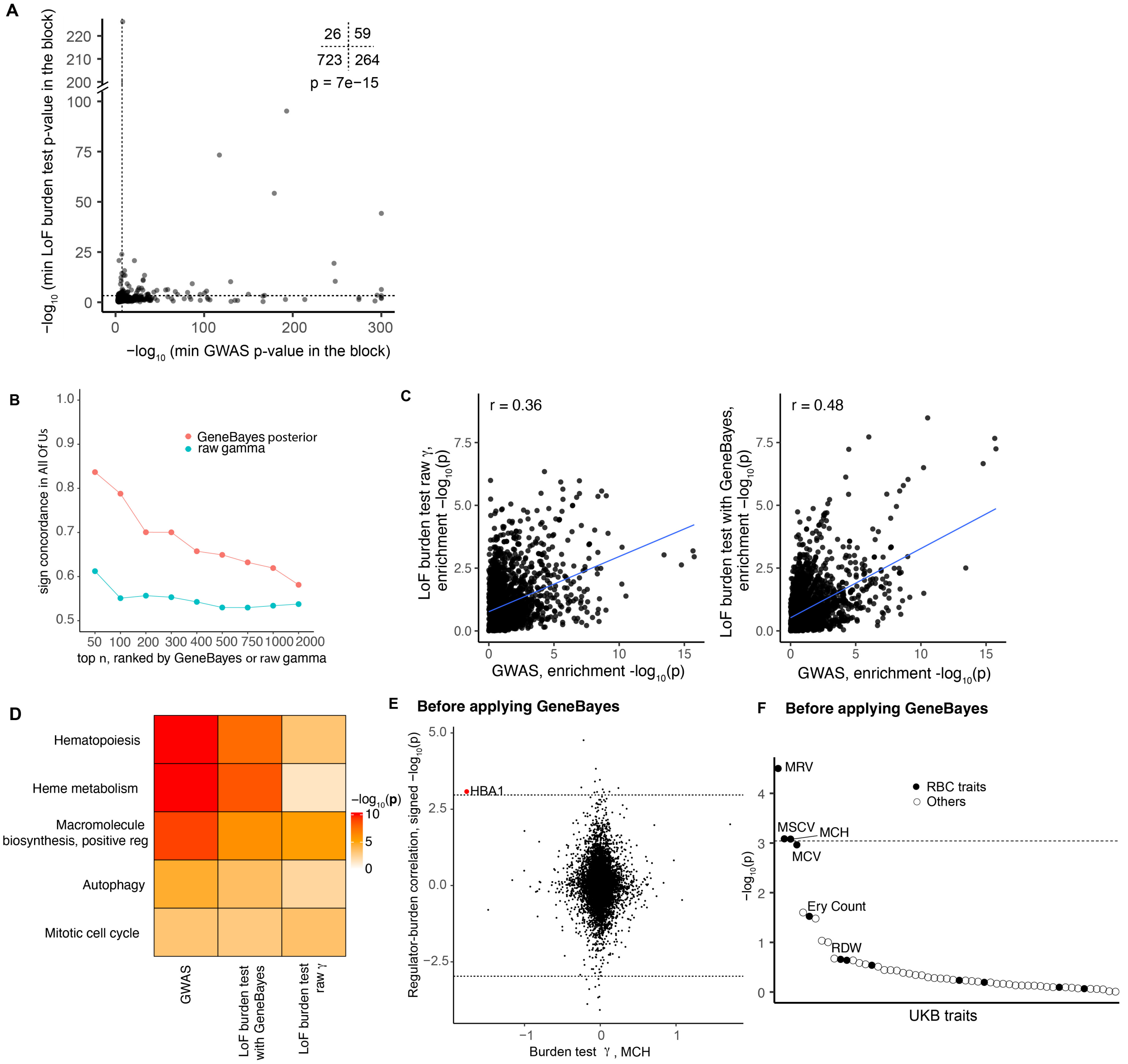

Figure S3: **Evaluation of GeneBayes, related to Figure 2 and 3.** ***A)*** *Comparison of GWAS and LoF burden test associations for MCH. We took the minimum GWAS p-value within an LD block, and the minimum LoF burden test p-value for any gene that overlaps the LD block. Dotted lines indicate* $p = 5 \times 10^{-8}$ *for GWAS and* $p = 5 \times 10^{-4}$*, which corresponds to an FDR of 0.1 for the LoF burden test. Numbers of blocks in each quadrant are depicted on the top right corner. p-value is from a two-sided Fisher's exact test.* ***B)*** *Sign concordance of burden test top hits in All Of Us. The result is for RDW. We ranked the genes based on absolute burden test effect size in UKB, either before or after GeneBayes, and assessed the fraction of genes that had the same sign of associations in All Of Us.* ***C)*** *Enrichment of GO and MsigDb hallmark pathways for genetic associations in MCH, before (left) or after (right) GeneBayes. For GWAS, the closest genes to the lead hits were ordered by p-values. For the LoF burden test, whether or not GeneBayes was applied, genes were ordered by absolute effect sizes. For both GWAS and LoF, the top 200 genes were used for the enrichment analyses.* ***D)*** *Enrichment of top 200 genes from GWAS or LoF top hits with or without applying GeneBayes to representative pathways.* ***E)*** *Regulator-burden correlation for MCH is compared with their* γ *for MCH. Same comparison with Figure 3C, but this time using* γ *before applying GeneBayes. Dotted lines indicate the same threshold with Figure 3C.* ***F)*** *Correlation significance of HBA1 regulatory effects with gene effects across a variety of traits. Same comparison with Figure S4A, but this time using* γ *before applying GeneBayes. Dotted line indicates the same threshold with Figure S4A.*

bioRxiv preprint doi: https://doi.org/10.1101/2025.01.22.634424; this version posted January 24, 2025. The copyright holder for this preprint (which was not certified by peer review) is the author/funder, who has granted bioRxiv a license to display the preprint in perpetuity. It is made available under aCC-BY 4.0 International license.

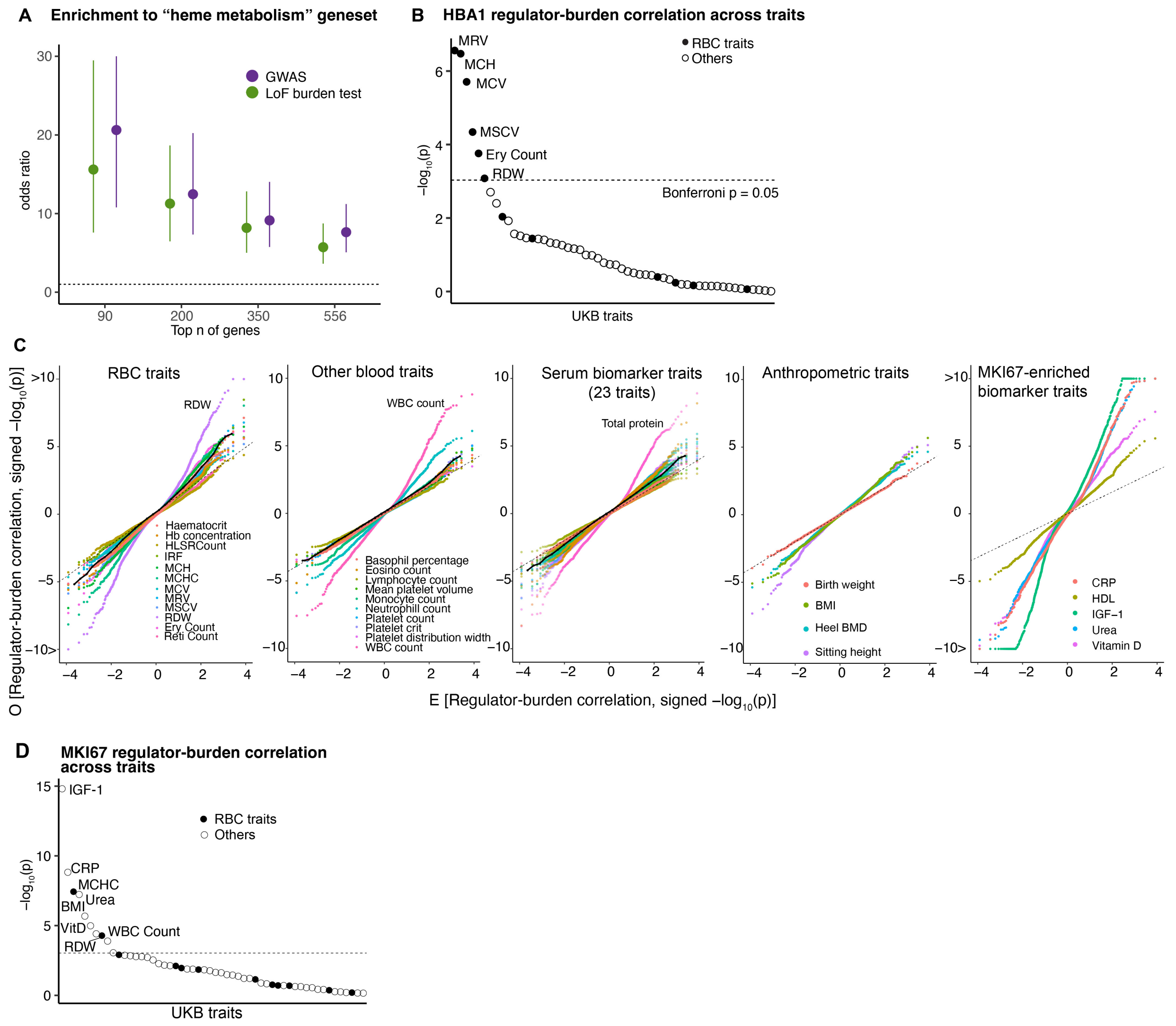

Figure S4: **Relevance of gene regulatory effects on trait associations, related to Figure 3.** ***A)*** *Enrichment of hemoglobin metabolism geneset for GWAS and LoF lead hits. For both GWAS closest genes and the LoF burden test, genes were ranked by association p-values, and top gene enrichment for the geneset was assessed using Fisher's exact test. Error bars indicate 95% confidence intervals.* ***B)*** *Correlation significance of HBA1 regulatory effects with gene effects (γ) across a variety of traits.* ***C)*** *Genome-wide QQ-plots for burden-regulator correlations for a wide variety of traits. Each dot indicates one gene. Black solid line indicates the median across each category of traits. For serum biomarker traits, 5 traits which showed extensive association with MKI67 regulatory effects are plotted separately.* ***D)*** *Correlation significance of MKI67 regulatory effects with gene effects across a variety of traits. Dotted line indicates the threshold for Bonferroni significance.*

bioRxiv preprint doi: https://doi.org/10.1101/2025.01.22.634424; this version posted January 24, 2025. The copyright holder for this preprint (which was not certified by peer review) is the author/funder, who has granted bioRxiv a license to display the preprint in perpetuity. It is made available under aCC-BY 4.0 International license.

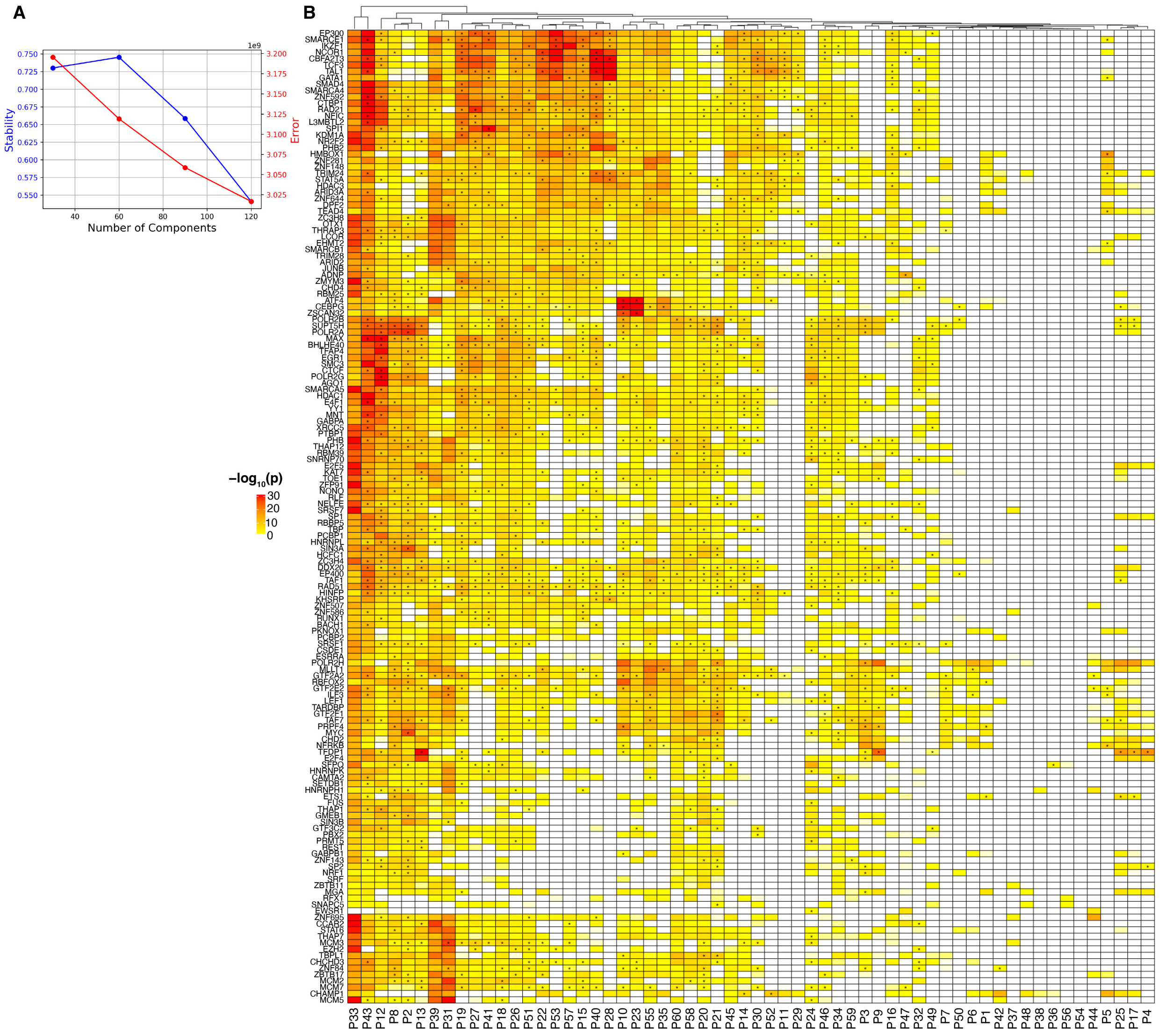

Figure S5: **Annotation of programs by transcription factor binding sites, related to Figure 4.** ***A)*** *Number of cNMF components against solution stability measured by the euclidean distance silhouette score of the clustering, and Frobenius error of the consensus solution, outputted by cNMF.* ***B)*** *Enrichment of transcription factor binding sites to program genes. Narrow peaks from ChIP-seq of transcription factors (TF) in K562 cells were used to calculate the enrichment (Methods). For significantly enriched TF-program pairs (FDR < 0.05), we tested the effect of knockdown of the TF on program activity and marked an asterisk if the KD also had an effect in the expected direction; that is, if the KD of an activator transcription factor decreased the program activity (p < 0.05), we marked it, and vice versa for repressor.*

bioRxiv preprint doi: https://doi.org/10.1101/2025.01.22.634424; this version posted January 24, 2025. The copyright holder for this preprint (which was not certified by peer review) is the author/funder, who has granted bioRxiv a license to display the preprint in perpetuity. It is made available under aCC-BY 4.0 International license.

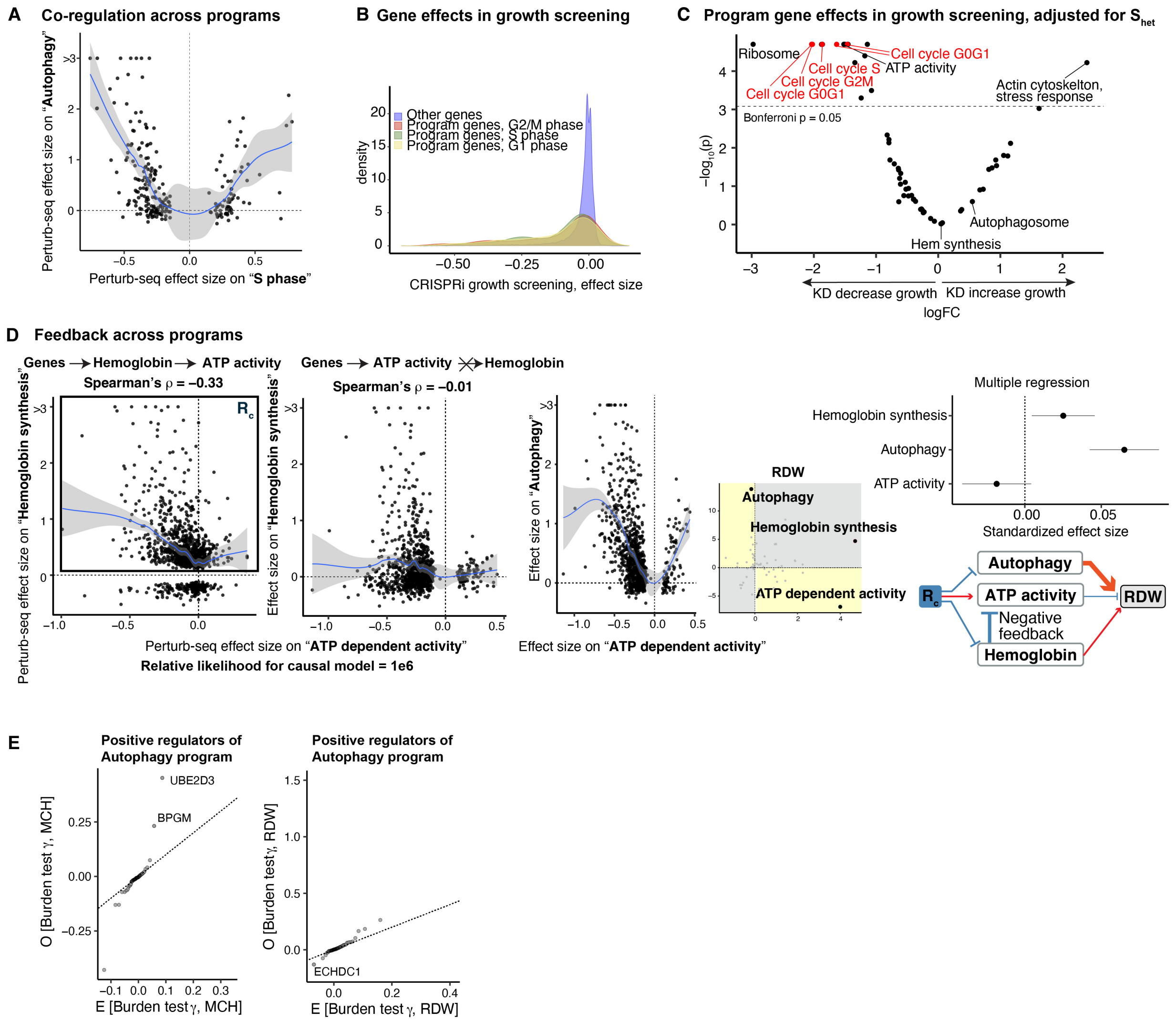

Figure S6: **Association of programs and regulators with traits, related to Figure 5.** *A) Co-regulation pattern between S phase and autophagy programs. Each dot is a gene that has significant regulatory effects on S phase program.* ***B)*** *Effects of cell cycle program genes KD on cellular growth. Growth screening data were obtained from an independent experiment using K562 [57]. The effect size is a normalized measure of the impact of KD on cellular growth compared to wild type, denoted as gamma in the original manuscript.* ***C)*** *Effects of program genes KD on cellular growth [57]. Here, for each program, we created 100,000 sets of control genes matched for S_{het} and compared the mean effects on cellular growth.* ***D)*** *Co-regulation pattern between ATP dependent activity, hemoglobin synthesis and autophagy programs. Genes with regulatory effects on hemoglobin program activity also had effects on ATP activity, but the opposite was not true. Right: Schematics for regulator association with the programs and trait.* ***E)*** *Distribution of burden test effect sizes for MCH (left) or RDW (right) for positive regulators of autophagy program. Note that the number of positive regulators is much smaller than that of negative regulators (Figure 5J).*

bioRxiv preprint doi: https://doi.org/10.1101/2025.01.22.634424; this version posted January 24, 2025. The copyright holder for this preprint (which was not certified by peer review) is the author/funder, who has granted bioRxiv a license to display the preprint in perpetuity. It is made available under aCC-BY 4.0 International license.

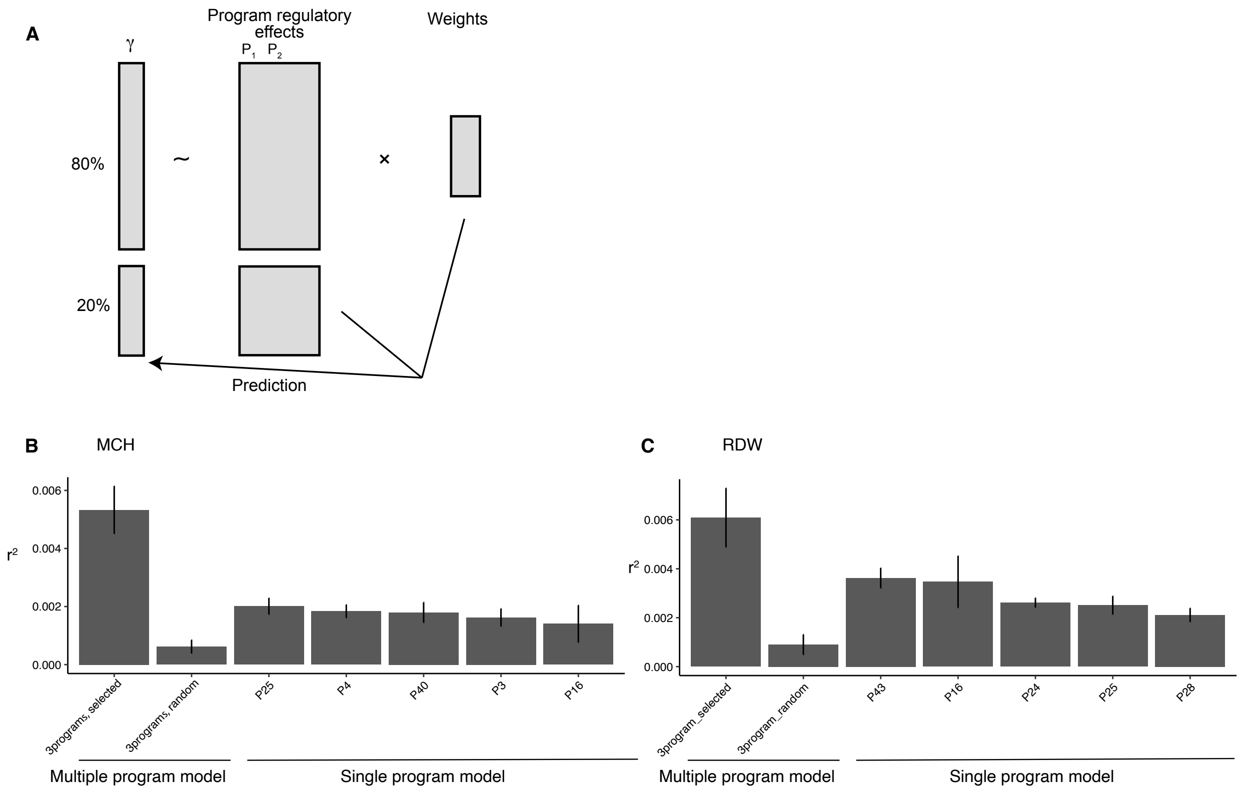

Figure S7: **Variance explained by multiple program regulators.** ***A)*** *We split the genes into a training set and a test set, and fitted multiple or single regression models to test the association between the gene regulatory effects on the program(s) and the gene effects on the trait (γ). We evaluated the variance explained by the model using the test gene set.* ***B-C)*** *Variance explained by the regression models for MCH (B) and RDW (C). "Programs, selected" refers to the programs selected from the regulator-burden correlations in the gene-to-program-to-trait map. "Programs, random" refers to the randomly selected sets of multiple programs. Single programs shown are the top 5 programs as to the variance explained. Error bars indicate* $1.96\times$ *standard errors.*

bioRxiv preprint doi: https://doi.org/10.1101/2025.01.22.634424; this version posted January 24, 2025. The copyright holder for this preprint (which was not certified by peer review) is the author/funder, who has granted bioRxiv a license to display the preprint in perpetuity. It is made available under aCC-BY 4.0 International license.

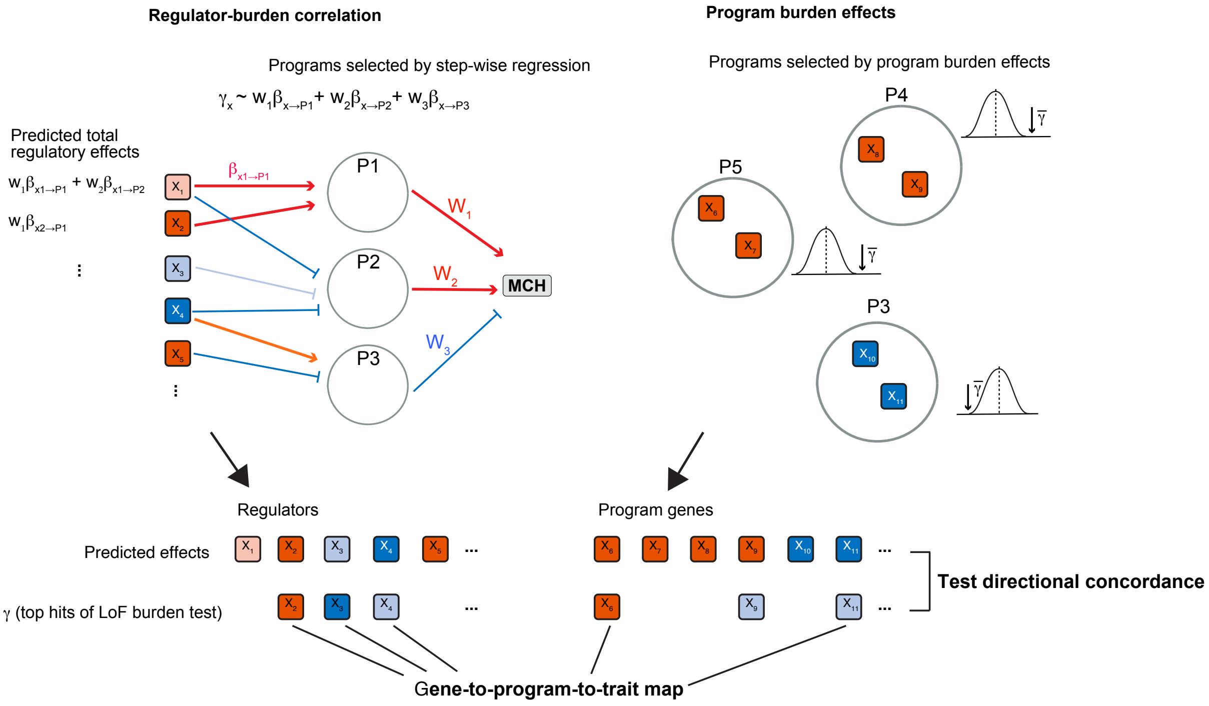

Figure S8: **Schematics for making multiple program association model.**

bioRxiv preprint doi: https://doi.org/10.1101/2025.01.22.634424; this version posted January 24, 2025. The copyright holder for this preprint (which was not certified by peer review) is the author/funder, who has granted bioRxiv a license to display the preprint in perpetuity. It is made available under aCC-BY 4.0 International license.

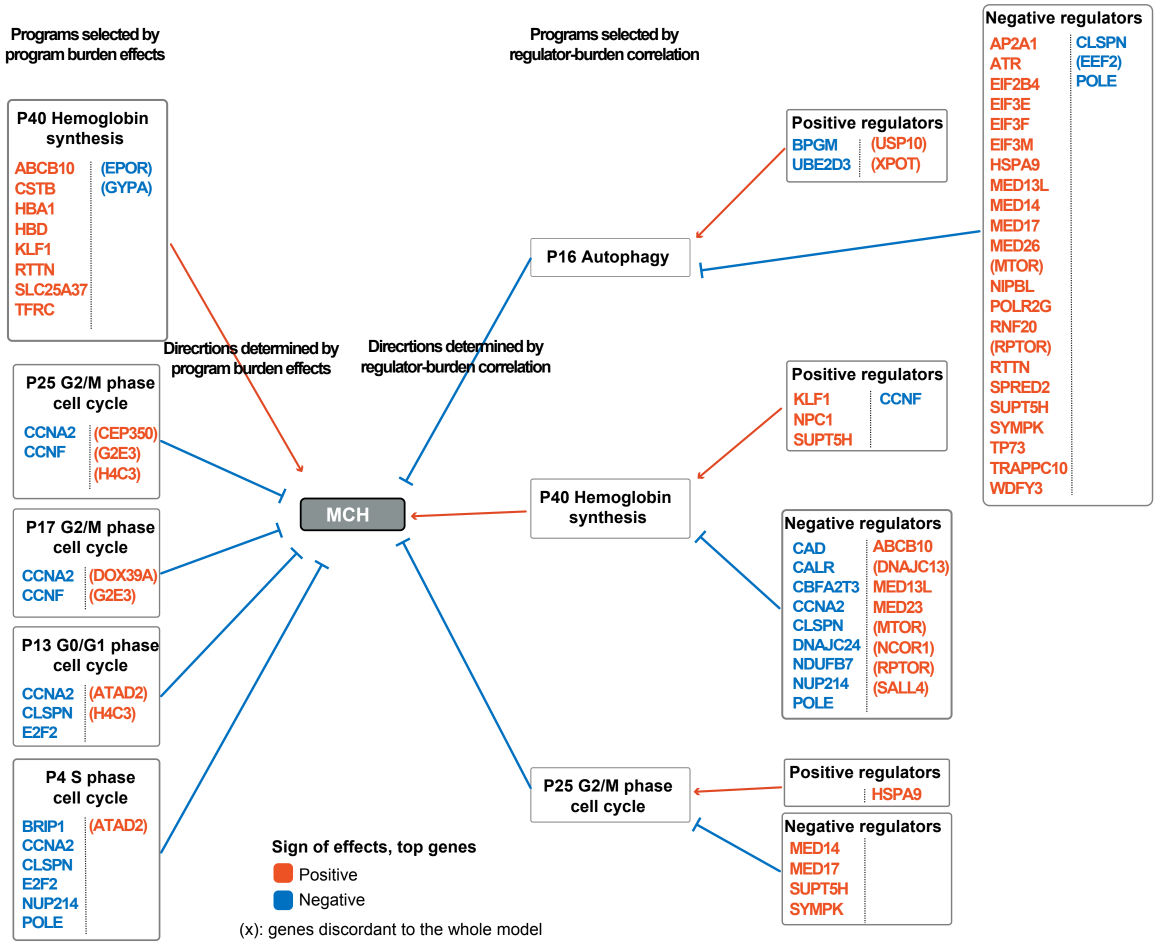

Figure S9: **Programs selected for modeling MCH associations.** *Programs were selected based on program burden effects (left) or regulator-burden correlations (right). For each program, top hits ($|\gamma| > 0.1$) for MCH that overlap with the top 200 loading genes (for program genes) or regulators (FDR < 0.05, for regulator genes) are listed. The color of genes correspond to the sign of γ. Genes in parentheses are discordant from the predicted directions from the overall model. Some of the genes are associated with multiple programs or regulators.*

bioRxiv preprint doi: https://doi.org/10.1101/2025.01.22.634424; this version posted January 24, 2025. The copyright holder for this preprint (which was not certified by peer review) is the author/funder, who has granted bioRxiv a license to display the preprint in perpetuity. It is made available under aCC-BY 4.0 International license.

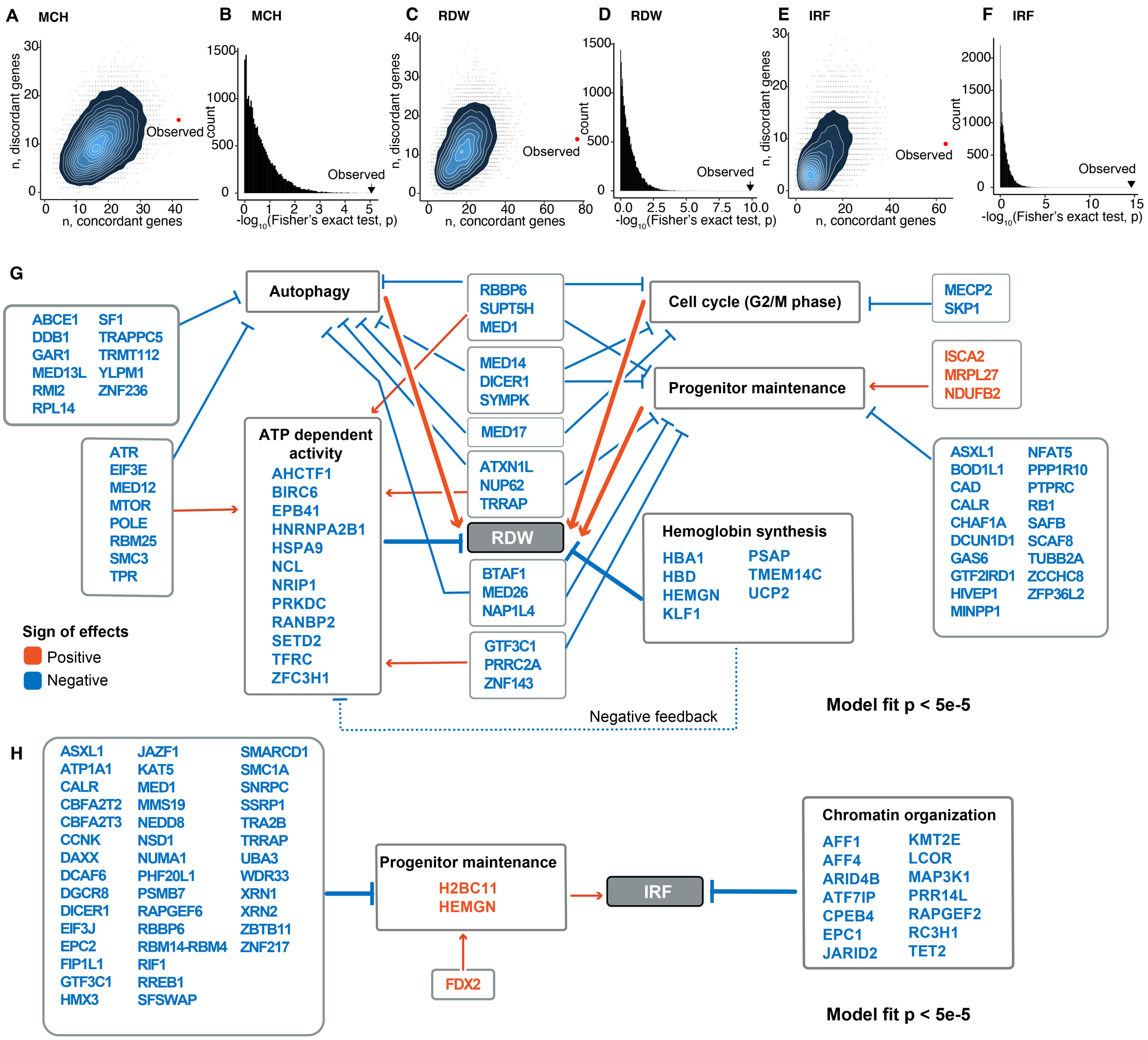

Figure S10: **Gene to program to trait maps, related to Figure 6.** *A) Number of top hits* $(|\gamma| > 0.1)$ *for MCH whose direction of associations were concordant or discordant with that predicted from the model. Grey points and their density plot are the results from 20,000 permutations. Red point shows the observed data.* ***B)*** *Distribution of top hits concordance p-values in permutation tests for MCH. In each permutation, we counted the number of top hits concordant with the model and evaluated its enrichment (Methods). The observed result showed the highest concordance compared to permuted sets.* ***C-F)*** *Same plots as (A) and (B), for RDW (C,D) and IRF (E, F).* ***G-H)*** *Gene to program to trait map for RDW (G) and IRF (H).*

bioRxiv preprint doi: https://doi.org/10.1101/2025.01.22.634424; this version posted January 24, 2025. The copyright holder for this preprint (which was not certified by peer review) is the author/funder, who has granted bioRxiv a license to display the preprint in perpetuity. It is made available under aCC-BY 4.0 International license.

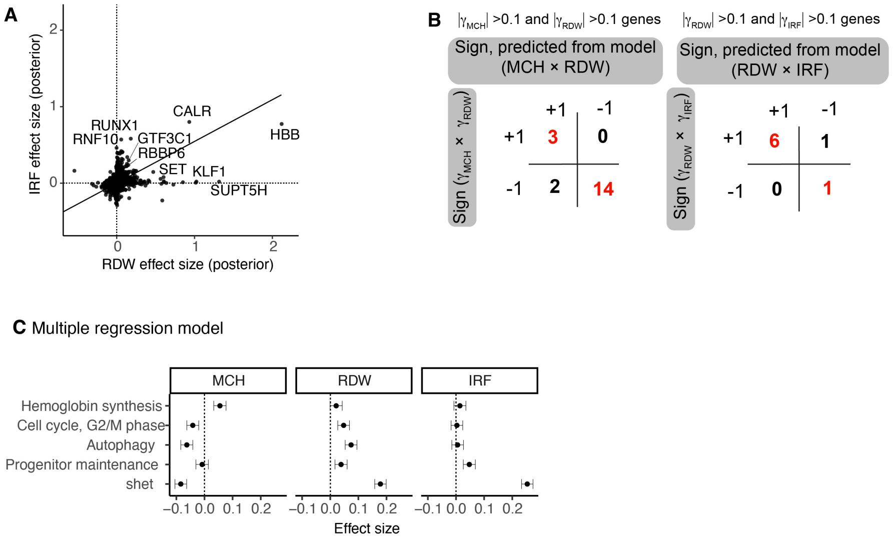

Figure S11: **Cross-trait comparisons of gene effects, related to Figure 6.** ***A)*** *Comparison of LoF burden test effect sizes after GeneBayes between IRF and RDW. The solid line corresponds to the first principal component.* ***B)*** *Cross-trait directional relationships of gene effects in the predicted gene-to-program-to-trait model and raw data from the LoF burden test. The left table shows the comparison between MCH and RDW, while the right table shows the comparison between RDW and IRF. For each table, only genes that have strong effects in both traits* ($|\gamma| > 0.1$) *and selected in the predicted model for both traits are considered. For instance, +1 means that the gene has strong effects for both traits in the same direction. MCH and IRF share few genes with strong effects and could not be compared.* ***C)*** *Correlation of regulatory effects on four programs or shet with* γ. *For each trait, correlation coefficients were estimated with the multiple regression model. Error bars indicate* 95% *CI.*

bioRxiv preprint doi: https://doi.org/10.1101/2025.01.22.634424; this version posted January 24, 2025. The copyright holder for this preprint (which was not certified by peer review) is the author/funder, who has granted bioRxiv a license to display the preprint in perpetuity. It is made available under aCC-BY 4.0 International license.

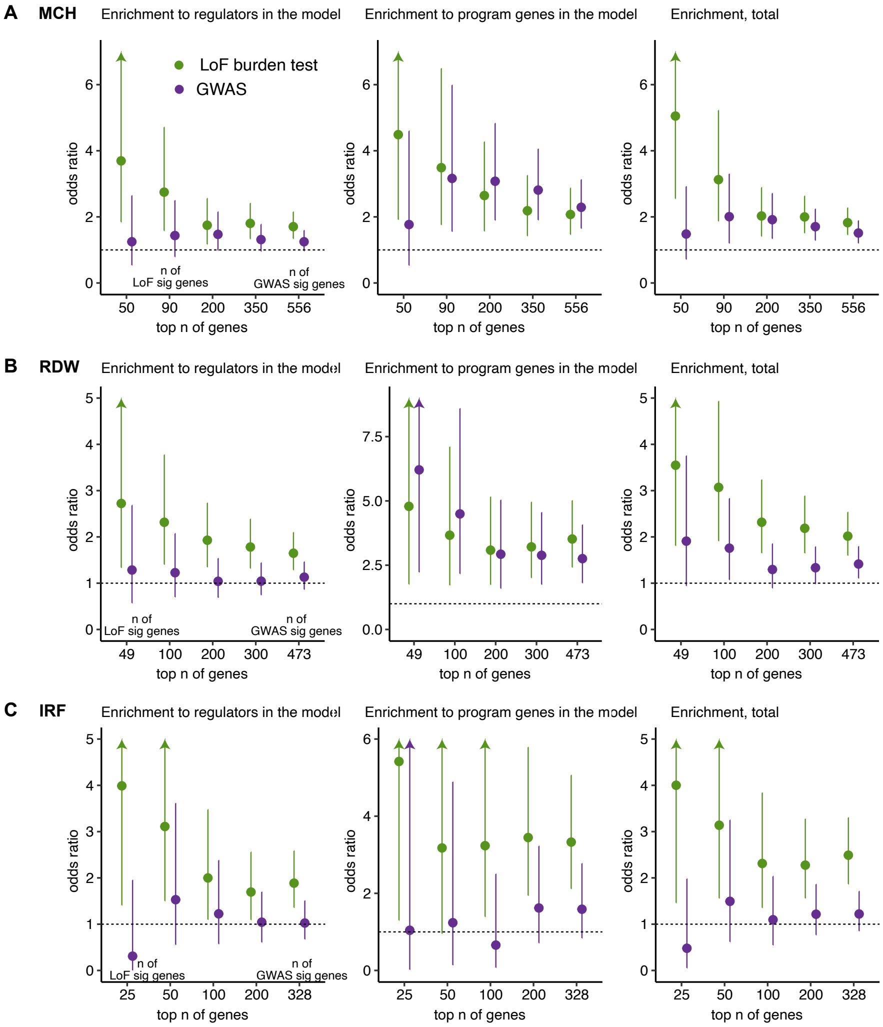

Figure S12: **Enrichment of GWAS and LoF top hits in the regulators and program genes in the model.** ***A-C)*** *From the gene-to-program-to-trait map for MCH (A), RDW (B) or IRF (C), we divided the genes into regulators, which have significant regulatory effects on the selected program, and program genes, which are among the top 200 loadings of the selected programs. We ranked the genes closest to the GWAS top hits based on p-values and the genes with LoF burden test based on absolute γ. Using different thresholds for top genes, we tested the enrichment in the genes selected by the model using Fisher's exact test. "Total" refers to the results for program and regulator genes combined.*

bioRxiv preprint doi: https://doi.org/10.1101/2025.01.22.634424; this version posted January 24, 2025. The copyright holder for this preprint (which was not certified by peer review) is the author/funder, who has granted bioRxiv a license to display the preprint in perpetuity. It is made available under aCC-BY 4.0 International license.

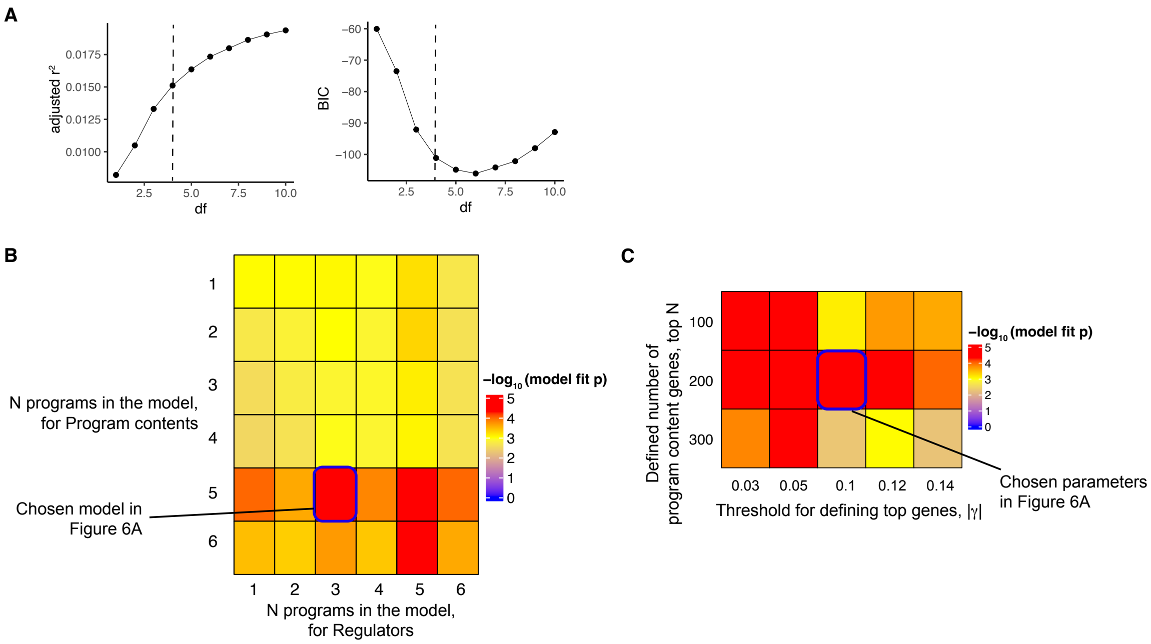

Figure S13: **Choice of parameters for gene-to-program-to-trait map.** ***A)*** *Adjusted* r^2 *and Bayesian Information Criterion (BIC) in the step-wise joint regression model. This is a result for selecting the number of programs whose regulators are associated with MCH. Dotted lines indicate the number of parameters chosen in the model.* ***B)*** *Model fit p-value, estimated from 20,000 permutations, for different numbers of programs included in the model for MCH. We varied the number of programs selected from regulator-burden correlations (x-axis) and program burden effects (y-axis) and evaluated the fit of the top hits to the model.* ***C)*** *Model fit p-value for different definitions of program genes or top hit genes from the permutation test. Here, we tested for MCH with the same number of programs in the final model.*
